# The Association Between Plant-Based Diet and Gallstone Disease: A Case-Control Study

**DOI:** 10.34172/mejdd.2025.430

**Published:** 2025-07-30

**Authors:** Mohammad Hossein Ebrahimizadeh, Ava Kheirizadeh, Azita Hekmatdoost, Moloud Ghorbani, Amir Sadeghi, Zahra Yari

**Affiliations:** ^1^Department of Clinical Nutrition and Dietetics, Faculty of Nutrition Sciences and Food Technology, National Nutrition and Food Technology Research Institute, Shahid Beheshti University of Medical Sciences, Tehran, Iran; ^2^Student Research Committee, Ahvaz Jundishapur University of Medical Sciences, Ahvaz, Iran; ^3^Department of Nutrition, Faculty of Medicine, Mashhad University of Medical Sciences, Mashhad, Iran; ^4^Research Institute for Gastroenterology and Liver Diseases of Taleghani Hospital, Shahid Beheshti University of Medical Sciences, Tehran, Iran; ^5^Department of Nutrition Research, National Nutrition and Food Technology Research Institute and Faculty of Nutrition Sciences and Food Technology, Shahid Beheshti University of Medical Sciences, Tehran, Iran

**Keywords:** Gallstone disease, Plant-based diet, Inflammation, Oxidative stress

## Abstract

**Background:**

Recent studies have emphasized the role of plant-based diets in reducing the risk of gallstone disease (GD) by reducing oxidative stress and inflammation. This study aimed to investigate the association between plant-based diet indices and GD risk.

**Methods:**

189 patients with newly diagnosed GD and 342 healthy controls participated in this case-control study. To assess overall adherence to a plant-based diet, three indices were calculated based on dietary data from the food frequency questionnaire: the plant-based dietary index (PDI), the healthful PDI (hPDI), and the unhealthful PDI (uhPDI). The association between plant-based diet indices and the risk of GD was assessed using logistic regression models.

**Results:**

A significant and inverse association was observed between PDI and the risk of GD, with an odds reduction of 48% in the crude model, 56% when adjusted for age and sex, and 59% when adjusted for additional confounders. Similar results were obtained for hPDI. Increasing the hPDI was associated with a 53% (in the crude model) to 67% (in the full adjusted model) reduction in the odds of GD. While increasing uhPDI was associated with an increased odds of GD. In the crude model, the highest uhPDI score increased the odds of the disease by 70%, and in the final model, the increase in odds reached 2.2-fold.

**Conclusion:**

Our study revealed that a healthy plant-based diet is associated with a reduced risk of GD, whereas an unhealthy plant-based diet may contribute to a greater susceptibility to disease, emphasizing the importance of diet quality in plant-based nutritional approaches. Further studies are needed to confirm these findings.

## Introduction

 Gallstone disease (GD), or cholelithiasis, is a hepatobiliary condition that affects 10% to 20% of adults worldwide and is associated with the most substantial socioeconomic complications.^1^ Moreover, a recent analysis of National Health Service data revealed a 72.2% increase in the total number of cholecystectomies performed annually between 2000 and 2019.^2^ Due to this condition, the United States incurs an annual direct cost of $5.8 billion.^3^ In a study conducted on cadavers, the prevalence of GD in Iran was reported to be 6.3%.^4^ Age above 40, genetic predisposition, ethnic background, female sex, and being pregnant are all non-modifiable risk factors for the development of gallstones. Other modifiable risk factors include obesity, diabetes, metabolic syndrome, extreme weight loss, high-calorie food consumption, some medications, smoking, and inactive lifestyle.^5,6^

 According to nutritional epidemiologists, assessing dietary patterns rather than focusing on individual food components can better reveal the relationship between diet and health status.^7^ Several studies have shown that diets rich in animal proteins, carbohydrates, and cholesterol increase the risk of GD, while diets high in vegetables, fruits, and total fiber reduce the risk.^8^ In this regard, new indices have been proposed, including the plant-based dietary index (PDI), the healthful PDI (hPDI), and the unhealthful PDI (uhPDI). The PDI assesses overall adherence to a plant-based diet, and the hPDI measures higher adherence to healthy plant foods (such as fruits, vegetables, whole grains, nuts, and legumes) and lower adherence to unhealthy plant foods (refined grains and foods high in sugar), whereas the uhPDI scores the opposite. In all three indices, animal foods receive a negative score.^9^

 To the best of our knowledge, there have been limited studies published on the association between the risk of gallstone formation and plant-based dietary indices. For the first time, in this case-control study, we evaluated whether adherence to a healthful versus an unhealthful version of a plant-based diet was associated with the risk of GD occurrence in Iranian adults.

## Materials and Methods

###  Study Design and Population

 The methodology of this study has been fully described elsewhere.^10^ Here, additional explanations are provided briefly. Between November 2017 and October 2018, this case-control study was conducted at Taleghani Hospital in Tehran, Iran. The cases involved patients with gallstones or common bile duct (CBD) stones, confirmed by ultrasonography, for a period of one month or less. The age- and sex- matched control group was composed of patients who were admitted to the other departments of the same hospital and did not have a history of GD, CBD, or hepatic conditions that were confirmed by ultrasonography. Participants had to be 18 years or older and willing to participate in the research program. Patients with a history of autoimmune conditions, malignancies, and inflammatory or infectious conditions, as well as pregnant or lactating mothers, were also excluded. Of the total 617 patients studied, 66 were ineligible and 551 patients were included in the study, 20 of whom were excluded due to incomplete data. Finally, 342 controls and 189 cases were retained. Each participant completed a written informed consent form. The Ethics Committee of the Research Institute of Gastroenterology and Liver Diseases (IR.SBMU.RIGLD.REC.1396.159) approved the protocol of the study.

###  Dietary Assessment and PDI, hPDI, and uhPDI Calculations 

 A reliable and valid semi-quantitative food frequency questionnaire (FFQ) was used to assess the food consumption of subjects during the year preceding their gallstone diagnosis in cases, as well as hospital admissions in the control group. Although recall bias is inevitable in FFQ, to reduce it, an experienced nutritionist was instructed to complete the questionnaire, and a food picture album was also provided to the patients to estimate sizes and quantities. The interviewer asked the subjects to indicate their daily, weekly, monthly, or annual consumption frequency for each food item, and then the obtained data were converted to grams. The Nutritionist IV software was used to analyze the collected data. The United States Department of Agriculture (USDA) provided the Food Composition Table (FCT), which we used to calculate the energy and nutritional values.

 Three plant-based dietary indices were calculated, including PDI, hPDI, and uhPDI, following the methodology outlined by Satija et al.^11^ Eighteen groups of foods were established to represent both healthy and unhealthy plant-based foods, with categorizations derived from the existing literature regarding their associations with chronic diseases (see Supplementary Table S1 from Satija).^11^ These food groups were split into quintiles of consumption, with each quintile being assigned a score ranging from 1 to 5. In the setting of the PDI, participants were assigned a score of 5 for each plant food group if their consumption was in the highest quintile, a score of 4 for the second-highest quintile, and so forth, which eventually resulted in a score of 1 for consumption in the lowest quintile (indicating positive scores). Also, participants in the highest quintile of animal-based food groups were assigned a score of 1, a score of 2 for consumption in the second-highest quintile, and so forth. A score of 5 was given for consumption in the lowest quintile, indicating reverse scores.

 For the hPDI, positive scores were assigned to healthy plant-based food groups, while negative scores were assigned to less healthy plant-based foods and animal-based foods. In contrast, for the uhPDI, positive scores were assigned to less healthy plant-based foods, while reverse scores were assigned to healthy plant-based foods and animal-based foods. Finally, these three indices were divided into tertiles to examine their associations with the risk of gallstone formation.

###  Statistical Analysis

 The statistical analysis was conducted using the SPSS software version 19 (SPSS Inc., Chicago, Illinois). We assessed the normality of the variables via a histogram and the Kolmogorov-Smirnov test. This research presents the baseline characteristics and dietary intakes of participants, expressed as means ± SD for quantitative variables and as percentages (%) for qualitative variables. Participants were divided into three tertiles based on PDI, hPDI, and uhPDI scores and compared using a one-way analysis of variance (ANOVA) for normally distributed variables and chi-square for categorical parameters, respectively. Crude and multivariable-adjusted models of logistic regressions were used to illustrate the probabilities of GD across each tertile of the dietary indices: PDI, hPDI, and uhPDI. The adjusted model accounted for confounding factors, including age, sex, BMI, caloric intake, physical activity, smoking, and alcohol use.

## Results

Table 1 presents the general characteristics of the study participants across tertiles of PDI, hPDI, and uhPDI. Individuals with higher PDI scores tended to be younger, more frequently female, less likely to smoke, taller, had higher caloric intake, lower BMI, and higher hPDI scores, along with lower uhPDI scores. Similarly, participants with the highest hPDI scores were generally younger, more often male, less likely to smoke, appeared taller, were more physically active, had higher body mass index (BMI), and also showed higher PDI and hPDI scores with lower uhPDI scores. Conversely, those with higher uhPDI scores were older, more likely to be male, exhibited higher smoking rates, were taller, and had lower PDI and hPDI scores, but higher uhPDI scores.

**Table 1:** Characteristics of study participants according to the tertiles of PDI, hPDI, and uhPDI

	**Tertile of total PDI**			
	**T1**	**T2**	**T3**	***P*** ** value**
Men, %	49.7	47	17.2	< 0.001
Age (y)	55.1 ± 14.6	54.6 ± 12.5	48.9 ± 11.7	< 0.001
IPAQ level, %				0.283
1	80	72	74	
2	18	24	22	
3	2	4	4	
Alcohol drinker, %	2.3	2.7	2.4	0.959
Smoker, %	20	16.4	7.7	0.004
Weight, kg	71.7 ± 14.7	73.8 ± 13.8	74.1 ± 11.6	0.196
Height, cm	165.1 ± 9.4	165.8 ± 8.8	163.2 ± 7.9	0.015
Body mass index, kg/m^2^	26.3 ± 4.8	26.8 ± 4.3	27.8 ± 3.7	0.004
Calorie intake (kcal/day)	2193 ± 593	2459 ± 610	2426 ± 626	< 0.001
PDI	53.6 ± 1.9	58.5 ± 1.3	66.3 ± 4	< 0.001
hPDI	60 ± 2.9	63 ± 3.2	67.8 ± 3.9	< 0.001
uPDI	76.5 ± 2.3	75.2 ± 3.1	71.3 ± 4.4	< 0.001
	**Tertile of total hPDI**			
	**T1**	**T2**	**T3**	**P value**
Men, %	46.5	58.7	83.5	< 0.001
Age (y)	52.5 ± 15.6	54.6 ± 11.2	50.9 ± 12.2	0.035
IPAQ level, %				0.005
1	84	71	68	
2	15	26	26	
3	1	3	6	
Alcohol drinker	2.9	2.8	1.9	0.819
Smoker, %	21	15	8	0.003
Weight, kg	72.6 ± 14.7	73.6 ± 13.1	73.9 ± 12.1	0.668
Height, cm	166.1 ± 9.4	165.2 ± 8.7	163.1 ± 7.8	0.006
Body mass index, kg/m^2^	26.9 ± 4.5	26.9 ± 4.2	27.8 ± 4	0.007
Calorie intake (kcal/day)	2364 ± 663	2388 ± 621	2292 ± 599	0.355
PDI	55.1 ± 3.3	58.7 ± 3.5	65.2 ± 5.3	< 0.001
uPDI	58.8 ± 1.9	63.3 ± 1.2	69.2 ± 2.8	< 0.001
uPDI	76.6 ± 2.7	74.9 ± 3.2	70.1 ± 3.8	< 0.001
	**Tertile of total uhPDI**			
	**T1**	**T2**	**T3**	**P value**
Men, %	12.4	48.1	52.8	< 0.001
Age (y)	49.6 ± 12.1	53.1 ± 12.8	55.8 ± 14.2	< 0.001
IPAQ level, %				0.422
1	73	74	79	
2	22	23	19	
3	5	3	2	
Alcohol drinker	0.6	2.7	4	0.122
Smoker, %	7	18.6	18.2	0.003
Weight, kg	72.5 ± 11.8	73.6 ± 14.7	73.5 ± 13.6	0.711
Height, cm	162.2 ± 7.5	166.4 ± 9.3	165.4 ± 8.9	< 0.001
Body mass index, kg/m^2^	27.5 ± 3.9	26.5 ± 4.8	26.9 ± 4.6	0.082
Calorie intake (kcal/day)	2341 ± 624	2338 ± 573	2405 ± 646	0.528
PDI	64.3 ± 5.8	57.8 ± 4.4	56.4 ± 3.6	< 0.001
hPDI	67.6 ± 4.3	62.9 ± 3.3	60.4 ± 3.1	< 0.001
uPDI	69.5 ± 2.8	75.1 ± 1	78.3 ± 1.2	< 0.001

PDI: plant-based diet index, hPDI: healthful plant-based diet index, uhPDI: unhealthful plant-based diet index Values are means ± SDs for continuous variables and percentages for categorical variables. ANOVA for quantitative variables and χ2 test for qualitative variables.

Table 2 presents multivariable-adjusted odds ratios (ORs) and 95% confidence intervals (CIs) for GD across tertiles of the dietary indices, including PDI, hPDI, and uhPDI. The crude model revealed a significant reverse association between the PDI (48% lower) and hPDI (53% lower) scores and the odds of GD. After adjustment for confounding factors, including age, sex, energy intake, BMI, physical activity, smoking, and alcohol intake, this association was strengthened. Also, in the crude model, an increase in uhPDI score was associated with a 70% increase in the odds of developing GD. After adjusting for age and sex, this association became stronger (OR = 2.1; 95% CI: 1.2-3.5, P trend = 0.001). Additionally, in the multivariable-adjusted model (Model 3), this association remained significant (OR = 2.2; 95% CI: 1.3-3.5, *P *trend = 0.003).

**Table 2:** Odds and 95% confidence interval for the occurrence of the gallstone according to the tertiles of PDI, hPDI, and uhPDI

	**Tertiles of PDI**			***P*** ** trend**
	**T1 (<56.3)**	**T2 (56.3-61)**	**T3 (61≤)**	
No. of cases	86	58	45	< 0.001
Model 1	ref	0.61 (0.39-0.93)	0.52 (0.33-0.81)	0.004
Model 2	ref	0.58 (0.38-0.91)	0.44 (0.27-0.71)	0.001
Model 3	ref	0.62 (0.38-1)	0.41 (0.24-0.69)	0.001
	**Tertiles of hPDI**			
	**T1 (<61.2)**	**T2 (61.2-65.5)**	**T3 (65.5≤)**	
No. of cases	43	60	86	< 0.001
Model 1	ref	0.43 (0.27-0.67)	0.47 (0.3-0.74)	0.001
Model 2	ref	0.32 (0.2-0.52)	0.29 (0.17-0.49)	< 0.001
Model 3	ref	0.36 (0.21-0.58)	0.33 (0.2-0.6)	< 0.001
	**Tertiles of unPDI**			
	**T1 (<73.3)**	**T2 (73.3-76.5)**	**T3 (76.5≤)**	
No. of cases	43	61	85	< 0.001
Model 1	ref	1.3 (0.46-4.6)	1.7 (1.1-2.6)	0.012
Model 2	ref	1.2 (1.1-1.4)	2.1 (1.2-3.5)	0.001
Model 3	ref	1.47 (0.41-5.3)	2.2 (1.3-3.5)	0.003

PDI: plant-based diet index, hPDI: healthful plant-based diet index, uhPDI: unhealthful plant-based diet index Based on multiple logistic regression model. Model 1: crude. Model 2: adjusted for age and sex. Model 3: additionally adjusted for energy intake, BMI, physical activity, smoking, alcohol.

## Discussion

 There is no doubt that diet is a critical factor in the development of GD. This article aimed to investigate the association between a plant-based diet and GD. For a better understanding of the level of adherence to a healthy plant-based diet, three indices, including PDI, uPDI, and hPDI were determined. According to our results, higher PDI and hPDI contribute to lower GD odds, and a higher uhPDI score was associated with higher odds of GD. These associations persist after adjusting for co-factors.

 A recent study of 5673 participants from NHANES (National Health and Nutrition Examination Survey) showed that a higher uPDI score was associated with a 53% increased risk of GD but failed to show a significant association between PDI, hPDI, and GD.^12^ One of the most important differences between this study and ours was the use of a 24-hour food recall instead of an FFQ, which may not fully reflect the participants’ usual dietary intake and cannot account for daily variations. Several studies have examined plant-based diets, such as vegetarian diets and the risk of GD, and conflicting results have been reported. A Taiwanese study compared the risk of symptomatic GD in vegetarians versus non-vegetarians, involving 4839 people, and showed that a vegetarian diet was associated with a reduced risk of GD in women, but not in men.^13^ In contrast, a cohort study of British vegetarians and non-vegetarians, comprising 49,652 adults followed for 13.85 years, revealed a small but significant positive association between a vegetarian diet and symptomatic GD.^14^ The researchers stated that since cholecystokinin (CCK), which plays a crucial role in gallbladder contraction, is stimulated to a lesser extent by fats and carbohydrates, it appears that consuming less fat may lead to increased gallbladder stasis by reducing CCK stimulation. Vegetarian diets appear to vary across populations and cultures, with East Asians typically consuming fruits and vegetables rich in fiber, antioxidants, and phytochemicals (higher hPDI scores). However, European vegetarian diets tend to be higher in starch (higher uhPDI scores). This could explain the different findings of the studies.

 Although the pathogenesis of the disease and the mechanisms of its association with a plant-based diet are not wholly understood, studies have, to a limited extent, examined existing hypotheses. Some of the proposed mechanisms include: insulin resistance,^1,15^ increased hepatic cholesterol synthesis,^1^ serum LDL-C level,^1,16^ inflammatory status,^17^ obesity,^1,15^ hyper-secretion of cholesterol into bile,^1^ and adherence to an unhealthy diet (high uhPDI score) with a high intake of refined grains and starches,^18,19^ high-cholesterol and high-fat foods.^20^ The association of a plant-based diet with some of these risk factors has been investigated, showing promising results in reducing the risk of GD following a high-PDI diet. In a study by Chang et al,^13^ it was shown that a plant-based diet can reduce serum cholesterol, which in turn is associated with a reduced risk of GD. The researchers also showed that a plant-based diet is more effective in those with normal cholesterol levels and in women. This is imperative because the prevalence of GD in women is about twice as high as in men. Another possible mechanism for the association of plant-based diets with reduced risk of GD is their high fiber content. Fiber may decrease cholesterol absorption by reducing intestinal transit time, thereby reducing the risk of GD.^21,22^

 In a 2019 study, Cortés and colleagues examined the association between insulin resistance (IR) and the risk of GD and proposed several mechanisms.^15^ IR appears to cause GD by increasing hepatic cholesterol secretion into bile and cholesterol saturation. IR also reduces bile acid synthesis by inhibiting enzymes of the bile acid biosynthesis pathway. On the other hand, hypomotility of the gallbladder due to IR can cause cholesterol crystallization and stimulate gallstone formation. IR is also associated with metabolic dysfunction–associated steatotic liver disease, which is strongly associated with GD. Both conditions share the same metabolic pathways.^15^ Recent research has shown that plant-based or vegetarian diets can improve IR.^19,23^ The primary reasons a plant-based diet can reduce IR include weight loss, decreased visceral fat, lower saturated fat content, and increased fiber, antioxidant, and plant sterol content.^24^

 Inflammation is a major factor in the pathogenesis of GS, as in many diseases.^25-27^ Inflammatory conditions impair the gallbladder’s ability, such as pH regulation, absorption, and contraction. Also, inflammation stimulates cholesterol crystallization through the stimulation of mucin and immune-related proteins. For instance, hs-CRP is thought to cause thickening of the gallbladder wall and decrease its motility, which in turn is associated with impaired bile emptying and gallstone formation.^26^

 Various studies have pointed to the role of a healthy plant-based diet in reducing inflammation and related diseases.^28-30^ The fiber content of plant-based diets appears to play a role in this association. On the one hand, dietary fiber improves the intestinal barrier by modifying the gut microbiota and reducing inflammatory biomarkers. On the other hand, fiber, by reducing energy density, can help alleviate obesity, which is a pro-inflammatory condition and a risk factor for GD. Additionally, plant-based diets are rich in phytochemicals and polyphenols, which act as anti-inflammatory agents and increase the body’s antioxidant capacity.^28-30^

 The limitations of the study should be considered when interpreting and generalizing the results. In this case-control study, the causal relationship between exposure and outcome is not clear. Additionally, the FFQ was used to collect dietary data, which is prone to recall bias; however, we adjusted the results accordingly. It is also worth noting that not all confounders can be adjusted for in the analyses.

## Conclusion

 Overall, the results indicated a significant inverse association between a healthy plant-based diet and a reduced risk of GD. Additional interventional and exploratory studies in larger populations are recommended to confirm and generalize the findings.

## References

[1] Sun H, Warren J, Yip J, Ji Y, Hao S, Han W (2022). Factors influencing gallstone formation: a review of the literature. Biomolecules.

[2] Lunevicius R, Nzenwa IC, Mesri M (2022). A nationwide analysis of gallbladder surgery in England between 2000 and 2019. Surgery.

[3] Sandler RS, Everhart JE, Donowitz M, Adams E, Cronin K, Goodman C (2002). The burden of selected digestive diseases in the United States. Gastroenterology.

[4] Farzaneh Sheikh Ahmad E, Tofighi Zavvareh H, Gharadaghi J, Sheikhvatan M (2007). Prevalence and characteristics of gallstone disease in an Iranian population: a study on cadavers. Hepatobiliary Pancreat Dis Int.

[5] Marschall HU, Einarsson C (2007). Gallstone disease. J Intern Med.

[6] Littlefield A, Lenahan C (2019). Cholelithiasis: presentation and management. J Midwifery Womens Health.

[7] Hu FB (2002). Dietary pattern analysis: a new direction in nutritional epidemiology. CurrOpinLipidol.

[8] Wirth J, Song M, Fung TT, Joshi AD, Tabung FK, Chan AT (2018). Diet-quality scores and the risk of symptomatic gallstone disease: a prospective cohort study of male US health professionals. Int J Epidemiol.

[9] Satija A, Bhupathiraju SN, Spiegelman D, Chiuve SE, Manson JE, Willett W (2017). Healthful and unhealthful plant-based diets and the risk of coronary heart disease in US adults. J Am Coll Cardiol.

[10] Neshatbini Tehrani A, Saadati S, Yari Z, Salehpour A, Sadeghi A, Daftari G (2023). Dietary fiber intake and risk of gallstone: a case-control study. BMC Gastroenterol.

[11] Satija A, Bhupathiraju SN, Rimm EB, Spiegelman D, Chiuve SE, Borgi L (2016). Plant-based dietary patterns and incidence of type 2 diabetes in US men and women: results from three prospective cohort studies. PLoS Med.

[12] Li L, Liu C, Xia T, Li H, Yang J, Pu M (2024). Association between plant-based dietary index and gallstone disease: a cross sectional study from NHANES. PLoS One.

[13] Chang CM, Chiu TH, Chang CC, Lin MN, Lin CL (2019). Plant-based diet, cholesterol, and risk of gallstone disease: a prospective study. Nutrients.

[14] McConnell TJ, Appleby PN, Key TJ (2017). Vegetarian diet as a risk factor for symptomatic gallstone disease. Eur J Clin Nutr.

[15] Cortés VA, Barrera F, Nervi F (2020). Pathophysiological connections between gallstone disease, insulin resistance, and obesity. Obes Rev.

[16] Zeng D, Wu H, Huang Q, Zeng A, Yu Z, Zhong Z. High levels of serum triglyceride, low-density lipoprotein cholesterol, total bile acid, and total bilirubin are risk factors for gallstones. Clin Lab 2021;67(8). doi: 10.7754/Clin.Lab.2021.201228. 34383399

[17] Meng C, Liu K (2023). Higher levels of systemic immune-inflammatory index are associated with the prevalence of gallstones in people under 50 years of age in the United States: a cross-sectional analysis based on NHANES. Front Med (Lausanne).

[18] Lysandra A, Wairooy N, Ifadha R, Sugiarto AA, Angelo Albright I, Izzah AF (2022). Risk factor of dietary habit with cholelithiasis. J Community Med Public Health Res.

[19] Naseri K, Saadati S, Asadzadeh-Aghdaei H, Hekmatdoost A, Sadeghi A, Sobhani SR (2022). Healthy dietary pattern reduces risk of gallstones: results of a case-control study in Iran. Int J Prev Med.

[20] Cheng J, Zhuang Q, Wang W, Li J, Zhou L, Xu Y (2024). Association of pro-inflammatory diet with increased risk of gallstone disease: a cross-sectional study of NHANES January 2017-March 2020. Front Nutr.

[21] Heaton KW (2000). Review article: epidemiology of gall-bladder disease--role of intestinal transit. Aliment PharmacolTher.

[22] Kaur J, Rana SV, Gupta R, Gupta V, Sharma SK, Dhawan DK (2014). Prolonged orocecal transit time enhances serum bile acids through bacterial overgrowth, contributing factor to gallstone disease. J Clin Gastroenterol.

[23] Chen P, Zhao Y, Chen Y (2022). A vegan diet improves insulin resistance in individuals with obesity: a systematic review and meta-analysis. DiabetolMetabSyndr.

[24] Kahleova H, Matoulek M, Malinska H, Oliyarnik O, Kazdova L, Neskudla T (2011). Vegetarian diet improves insulin resistance and oxidative stress markers more than conventional diet in subjects with Type 2 diabetes. Diabet Med.

[25] Luo Y, Gao X, Xiao M, Yang F, Zhu X, Qiao G (2024). Association between dietary inflammatory index and gallstones in US adults. Front Nutr.

[26] Liu T, Siyin ST, Yao N, Duan N, Xu G, Li W (2020). Relationship between high-sensitivity C reactive protein and the risk of gallstone disease: results from the Kailuan cohort study. BMJ Open.

[27] Liu Z, Kemp TJ, Gao YT, Corbel A, McGee EE, Wang B (2018). Association of circulating inflammation proteins and gallstone disease. J Gastroenterol Hepatol.

[28] Bolori P, Setaysh L, Rasaei N, Jarrahi F, Yekaninejad MS, Mirzaei K (2019). Adherence to a healthy plant diet may reduce inflammatory factors in obese and overweight women-a cross-sectional study. Diabetes MetabSyndr.

[29] Thomas MS, Calle M, Fernandez ML (2023). Healthy plant-based diets improve dyslipidemias, insulin resistance, and inflammation in metabolic syndrome A narrative review. Adv Nutr.

[30] Craddock JC, Neale EP, Peoples GE, Probst YC (2019). Vegetarian-based dietary patterns and their relation with inflammatory and immune biomarkers: a systematic review and meta-analysis. Adv Nutr.

